# Encode a Letter and Get Its Location for Free? Assessing Incidental Binding of Verbal and Spatial Features

**DOI:** 10.3390/brainsci12060685

**Published:** 2022-05-24

**Authors:** Molly A. Delooze, Naomi Langerock, Robin Macy, Evie Vergauwe, Candice C. Morey

**Affiliations:** 1 School of Psychology, Cardiff University, 70 Park Place, Cardiff CF10 3AT, UK; deloozema@cardiff.ac.uk; 2 Faculty of Psychology and Science of Education, University of Geneva, Bureau 5158, 40 Boulevard Pont d’Arve, 1205 Genève, Switzerland; naomi.langerock@unige.ch (N.L.); evie.vergauwe@unige.ch (E.V.); 3 School of Philosophy, Psychology and Language Sciences, University of Edinburgh, 3 Charles Street, Edinburgh EH8 9AD, UK; robin.macy96@gmail.com

**Keywords:** working memory, short-term memory, spatial, verbal, binding

## Abstract

Previous studies have demonstrated that when presented with a display of spatially arranged letters, participants seem to remember the letters’ locations when letters are the focus of a recognition test, but do not remember letters’ identity when locations are tested. This strong binding asymmetry suggests that encoding location may be obligatory when remembering letters, which requires explanation within theories of working memory. We report two studies in which participants focused either on remembering letters or locations for a short interval. At test, positive probes were either intact letter–location combinations or recombinations of an observed letter and another previously occupied location. Incidental binding is observed when intact probes are recognized more accurately or faster than recombined probes. Here, however, we observed no evidence of incidental binding of location to letter in either experiment, neither under conditions where participants focused on one feature exclusively for a block, nor where the to-be-remembered feature was revealed prior to encoding with a changing pre-cue, nor where the to-be-remembered feature was retro-cued and therefore unknown during encoding. Our results call into question the robustness of a strong, consistent binding asymmetry. They suggest that while incidental location-to-letter binding may sometimes occur, it is not obligatory.

## 1. Introduction

Verbal–spatial binding, whether intentional (e.g., [1]) or incidental [2,3], is potentially revealing for working memory theory (e.g., [4,5]). Some models of working memory explicitly posit that verbal and non-verbal features are separately maintained (e.g., [6]), while others suggest that verbal and non-verbal memory phenomena must arise from interactions of different systems (e.g., [7]), and yet others posit that either sort of feature is ultimately maintained within a common attentional system [8]. Some work suggests that encoding one feature—for instance, letter identities—may not exclusively engage domain-specific processes. Ref. [2] report that, when presented with letters in locations, participants incidentally remembered irrelevant location information while focusing on verbal letter information, but not the reverse: when focusing on spatial location information, letters did not seem to be encoded along with them. This suggests that location information is uniquely predisposed to binding, in a way that letter information is not, a phenomenon that Elsley and Parmentier called *strong asymmetry*.

The observation of a strong asymmetry between letter and location information puts important constraints on the verbal–spatial separation in working memory for at least two reasons. First, although it was once believed that retaining the features of an encoded object was cost-free compared to encoding a simpler, single-feature object [9], it now seems fairly clear that encoding and maintaining multi-feature objects does come at an increased cost (e.g., [5,10,11]). However, several findings indicate that spatial location may be special, in that locations may get a free ride, at least briefly, when encoding other content. For example, when encoding color–shape combinations with an explicit instruction to ignore location, presenting objects in different locations at test impairs recognition, at least when the recognition probe occurs within a second or so from the presentation of the objects [12]. Likewise, Ref. [13] examined memory for colored squares and found that performance was best when the objects were presented in their original positions at test, again showing that original object-to-location bindings appear to persist for at least 1 s. Interestingly, when remembering spatially arranged letters, locations seem to be attached to letters for up to 15 s, even though irrelevant at test [2]. Thus, even though additional features typically come with a cost, this is often not the case for location information, regardless of the explicit relevance of remembering locations, an observation that [2] showed to be particularly robust when it concerns the location of letters.

Second, Elsley and Parmentier’s (2015) findings that location information comes for free when remembering letters’ locations, whereas letter information does not come for free when remembering locations, point to a strong asymmetry in terms of which working memory resources may be involved in each sort of memory task. However, in Elsley and Parmentier’s experiment, participants knew before each trial which feature they were required to remember on any given trial. Possibly, this may have incentivized them to inhibit irrelevant features, which could mean that the strong asymmetry they observed lies in differences in inhibiting the encoding of identity versus location, rather than to a strong asymmetry in underlying resources. Indeed, regarding the effects of verbal information on spatial memory and the reverse, it seems clear that task goals and demands provoke differences in how information is encoded, but precisely how regular and predictable these patterns are is not yet clear. Ref. [14] found that location recognition tests were slightly worse when participants tried to encode letter–location conjunctions while engaged in articulatory suppression, and [15] found that with sequentially presented lists, locations marked with phonologically similar letters were recalled worse than locations marked with dissimilar letters. Both of these outcomes show that there are circumstances in which a liability specific to the verbal domain somehow bleeds into the representation of spatial locations. This cannot be due only to motivating participants to explicitly remember letter–location binding, because Guérard and Tremblay’s task did not require that participants recalled the letters, only the sequence of locations where they were presented. On the other hand, path complexity, a manipulation known to impair spatial location recall [16,17], impacts verbal recall under some circumstances [18] but possibly not universally [19]. Taken together, whereas Elsley and Parmentier’s finding potentially indicates an important asymmetry in working memory resources underlying letter–location maintenance, other studies suggest that information is encoded or acted upon differently depending on the participants’ goal and various circumstantial factors. These factors potentially complicate the proposition that a strong asymmetry between verbal and spatial information in working memory is caused by obligatory location-to-letter binding, which makes it difficult to assess the implications of the strong asymmetry observed by [2] for working memory theories.

Because it is not yet clear under what circumstances location information might tag along when letters are encoded, we decided to reexamine the strong asymmetry between verbal and spatial information in letter–location binding maintenance in working memory. In particular, we set out to assess whether the incidental binding of location to letters depends on attention during encoding or, instead, toward internal processes during retention or retrieval. We do not know how much strong asymmetry depends on preventing untested, irrelevant information from being encoded, versus ignoring irrelevant details that one may happen to remember at test. We manipulated whether participants knew at encoding which feature would be tested by including trials in which probe features were pre-cued ahead of encoding, or alternatively retro-cued during the retention interval. In Elsley and Parmentier’s (2015) experiment, participants knew before each trial began which feature they were required to remember. This is similar to our condition using pre-cues. Possibly, in Elsley and Parmentier’s experiment, this may have incentivized the participants to inhibit irrelevant features, which could mean that the strong asymmetry lies in differences in inhibiting the encoding of identity versus location. In that case, we might only observe the asymmetric location-to-letter binding when test features are pre-cued. However, in retro-cued trials where the test feature was unknown at encoding, participants could not opt out of encoding both features and still perform well. Therefore, if we observe the obligatory encoding of location with letter features in retro-cued as well as pre-cued trials, then the processes leading to the cost-free encoding of locations with letters cannot be limited to attentional processes at encoding, thereby supporting the view that the strong asymmetry reflects an important asymmetry in working memory resources. Additionally, we aimed to explore the effect of set size on the observed asymmetry by manipulating whether three or five letter–location bindings were presented on any given trial. We anticipated that retro-cued trials would be more difficult than pre-cued trials; therefore, a set size of three was chosen to provide an easier condition, and we also included trials with a set size of five in case the easier three-item trials produced ceiling effects in some conditions. We chose five items instead of four to spread the levels by more than just a single value. In their paper, Elsley and Parmentier suggest that task difficulty may be a factor in determining binding asymmetry (e.g., Maybery, Leung, Terne, van Valkenburg & Parmentier, 2012, as cited in [2]). Possibly, the strong asymmetry is more prevalent when the verbal load is lower and attention is available to devote to locations. Alternatively, it may be the case that with fewer locations to remember, incidental binding emerges in both the letter and location memory tasks.

## 2. Experiment 1

### 2.1. Method

#### 2.1.1. Participants

Data were collected from 48 participants, who were students at the University of Edinburgh or Cardiff University. Participants completed the study voluntarily, and received course credits if relevant. Four participants were excluded from the final data set for prescribed medication use, due to the effect of some prescribed medications on memory and attentional focus. One participant’s data were excluded when it was discovered that they had been texting while participating, and another was excluded for an unusually slow mean response time (3.70 SD above average). The 42 remaining participants (33 female) ranged from 18 to 30 years old (*M* = 19.64, *SD* = 2.28).

#### 2.1.2. Apparatus and Materials

Participants performed the task in a private laboratory cubicle. The experimenter sat nearby to be available if needed. Stimulus presentation and response recording was controlled with custom software programmed in E-Prime 2.0 [20], which can be found on our Open Science Framework repository (https://osf.io/c9xzh/ (accessed on 1 January 2020)). Participants responded either via Psychology Software Tools response boxes or with the keyboard. Similar to Elsley and Parmentier’s (2015) design, letter stimuli were chosen randomly without replacement on each trial from a set of ten consonants (B, F, G, H, J, L M, Q, R, T). Letters were presented in their uppercase forms during the stimulus display and in their lowercase forms during the probe period. Likewise, spatial locations were chosen from a set of ten positions spaced evenly around the center of the screen in a circular formation.

#### 2.1.3. Procedure

**Figure 1:**
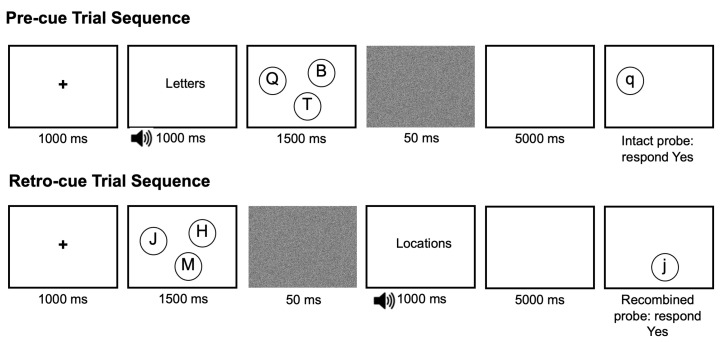
Trial events with timings for pre- and retro-cued trials. Negative probes, in which either the relevant feature or both features were new, were also included. Elements are not shown to scale. Reminder of feature test at probe screen appeared at the top of the screen, above the ranges of possible locations.

Participants performed two versions of the memory task, with a brief break in between. Each task began with a short practice block to confirm participants’ understanding of the instructions. Participants were not allowed to proceed with the experimental task until they had completed at least 6 out of the 8 practice trials correctly. Feedback was provided during practice, but not during the experimental trials. Figure 1 shows trial events and timings for both cue conditions. In each condition, participants were shown a fixation cross at the center of the screen for 1000 ms, followed by a display of three or five uppercase letters in circles for 500 ms per item (i.e., 1500 ms for three items and 2500 for five items). This was followed for 50 ms by a static visual pattern mask, then by a 5000 ms retention period.

In the pre-cued version of the task, participants observed a cue prior to presentation of the stimuli, letting them know whether letters or locations would be tested on that trial. Participants were shown a 1000 ms audio-visual cue before the stimulus display appeared. The cue consisted of a voice saying “letters” or “locations” accompanied by the word printed on the screen. Sound files were generated using the text-to-speech capability in MacOS, with the British-accented female voice “Kate” speaking the cue word “letters” or “locations”. At the end of each trial, they were then shown a single probe item along with a reminder of which feature was being tested. Participants were instructed to indicate whether the probed feature had appeared in the stimulus display. Participants were to respond as quickly and accurately as possible. The retro-cued task was identical to the pre-cued task, except that participants observed the cue to recall letters or locations after the stimulus display and mask, before the 5000 ms retention period began.

In both tasks, participants were instructed to answer “yes” if the cued feature on the test screen had been present in the stimulus array, disregarding the uncued feature. The to-be-ignored uncued feature varied systematically. The probes were either intact letter–location pairs (an old letter in the same location where it was previously presented), recombined pairs (e.g., an old letter in a different old location), new-letter and new-location (a new letter or location paired with an old feature) or both-new (neither the letter nor location had been in the stimulus display). Participants needed to answer “yes” to intact and recombined probes for the feature they had been cued to recall, and “no” to probes which contained a new feature in the tested dimension.

The ordering of the pre- and retro-cue tasks was counterbalanced. The number of items per stimulus display and the domain of the probe were randomized within each block.

### 2.2. Results

Our data and analysis scripts are available in our Open Science Framework repository (https://osf.io/c9xzh/, accessed on 1 January 2020). Our main analyses of interest were two Cue Timing (pre-cue vs. retro-cue task) by Cue Content (letters vs. locations) by Probe Type (intact vs. recombined probes) by Set Size (3 or 5 items) repeated-measures ANOVAs performed on accuracy and log-transformed response times to accurate probes. While negative probes must be included in the design for internal consistency, following the logic used previously in incidental letter–location binding research [2,3,21,22], incidental binding was determined by considering differences between intact and recombined positive probes. Observing slower or less accurate responses for recombined than intact probes indicates that the irrelevant feature is remembered and considered when making a response: if the irrelevant binding between letter and location is remembered, then intact probes should provoke a higher rate of correct responses than recombined probes, or correct identification of an intact probe should occur more quickly than a recombined probe.

#### 2.2.1. Accuracy

We first checked that the overall mean accuracy per participant at set size 3 was sufficiently high that we could believe that they completed the task sincerely. If randomly guessing, participants would achieve 50% correct, so we considered an average of 60% correct evidence of sufficient effort. One participant failed to reach this criterion, and was excluded from analyses.

**Figure 2:**
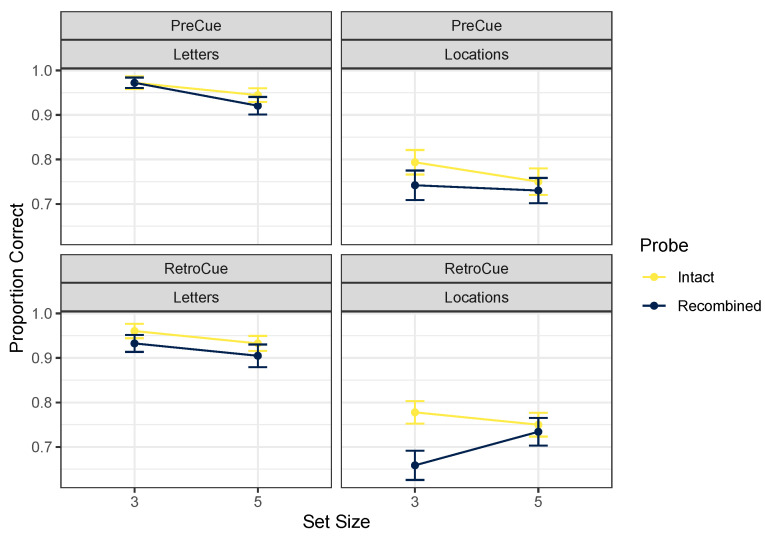
Mean proportions correct, Experiment 1. Error bars mark within-participant standard errors around the mean calculated with the Cousineau–Morey method [25].

Table 1 gives descriptive statistics for the full experimental design, and Figure 2 focuses on the intact and recombined trials to assess whether incidental binding occurred. Given that in the pre-cue version of the task, participants always knew which feature would be tested (as in [2]), we expected to observe a pattern consistent with the strong asymmetry hypothesis. This would mean that for letter tests, intact probes would be more likely to be correctly identified than recombined probes (reflecting the maintenance of irrelevant location information when letters were to be remembered), but for location tests, no difference should appear (reflecting the lack of maintenance of irrelevant letter information when locations were to be remembered). However, this pattern does not clearly emerge from Figure 2. The best model according to a Bayesian ANOVA [23,24] on proportions correct (after undergoing arcsine square-root transformation to adjust for non-equality of variances across conditions) included main effects of cue content and probe type, *BF* = $3.7\times 10^{51}$. Excluding an effect of set size was only marginally favored, *BF* = 1.65. Excluding the effect of cue timing was also only slightly favored, *BF* = 1.74. Including an effect of probe type was marginal, *BF* = 2.03, which means that in this analysis, it is not clear whether incidental binding occurred at all, although accuracy on intact probes was at least slightly better than for recombined probes in most conditions. Excluding an interaction between cue content and probe type was favored by a factor of 5.74. This outcome is not consistent with the strong asymmetry hypothesis that binding occurs when attending letters but not when attending locations. According to this analysis, intact probes were recognized somewhat more accurately regardless of whether letter or location was tested, and regardless of whether participants knew at encoding which feature would be tested.

#### 2.2.2. Response Times

We ran corresponding analyses on log-transformed response times, excluding incorrect responses. Response times were trimmed using the *trimr R* package [26], excluding correct responses more than 3 standard deviations from the per-participant mean.

**Figure 3:**
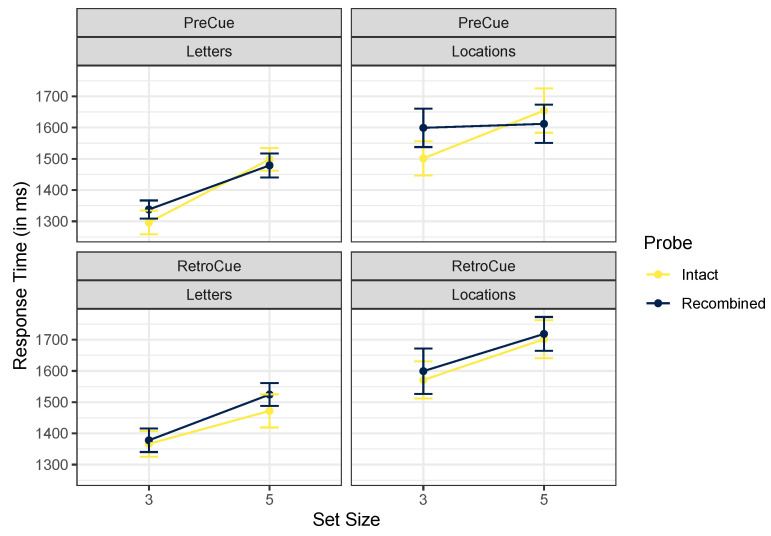
Mean trimmed reaction times, Experiment 1. Error bars mark within-participant standard errors around the mean calculated with the Cousineau–Morey method [25].

Table 2 gives descriptive statistics for the full experimental design, and Figure 3 presents the intact and recombined trials. Descriptively, response time results are consistent with accuracy results in that location judgments appear to be slower than letter judgments. Here, we see clear set size effects for both types of cued content. According to our Bayesian ANOVA, there was no evidence of incidental binding. The best model included only main effects of cue content and set size, *BF* = $2.9\times 10^{20}$. Excluding an effect of cue timing was slightly preferred, *BF* = 2.32. Excluding the crucial effect of probe type was favored by a factor of 22.54, and excluding the interaction between probe type and cue content was favored strongly, *BF* = 352.38. We therefore observed reasonably strong evidence *against* incidental binding in these data, with no evidence suggesting that incidental binding differed for letter versus location tests.

### 2.3. Discussion

Our results in this planned extension of [2] were inconsistent with the original findings, even in the most comparable conditions. We did not anticipate this: we firmly expected that at least when letter and location tests were pre-cued, we would observe patterns similar to those observed by Elsley and Parmentier, and could use this as a basis for interpreting results in the more novel retro-cued trials. One reason for this deviation could be that, unlike Elsley and Parmentier, we did not block letter and location probe tests. This could easily be done in the pre-cue condition. Indeed, a consistent block-wide instruction is conceptually the same as a pre-cue, which is why we expected to replicate their pattern. However, perhaps the differences between our findings are attributable to mixing letter and location trials. Perhaps the asymmetry Elsley and Parmentier that observed depends on instantiating an attentional set during encoding, and we did not observe it because our participants faced the added burden of revising their attentional set with each changing pre-cue. Alternatively, our participants may have adopted a strategy of encoding all features regardless of what was cued, but in a non-integrated fashion. Either way, our findings suggest, at the least, that location-to-letter binding is not necessarily obligatory.

We therefore followed up by running a closer replication of Elsley and Parmentier’s (2015) design. We abandoned the retro-cue condition, and instead implemented pre-cues in blocks in which letter and location tests were either randomly mixed (as in our Experiment 1) or purely composed of one type of test (as in Elsley and Parmentier). If the burden of updating one’s attentional set wipes out whatever resource or process leads to obligatory location encoding with letter, then we should see no evidence of this binding asymmetry for tests in the mixed-cue blocks, but replicate Elsley and Parmentier’s findings within the pure blocks.

## 3. Experiment 2

### 3.1. Method

In order to increase our chances of replicating Elsley and Parmentier’s (2015) findings, whilst also balancing an investigation into what factors may have caused the previous study to fail to replicate the expected effect, we made several small changes to the stimuli and procedure to match theirs: we changed the letter set to be identical, we abandoned our set size manipulation and always used 4-item displays, and we introduced a retention interval manipulation, including two of the intervals from their experiment. Rather than manipulating pre- and retro-cues, trials were organized in randomly ordered blocks: in mixed blocks, the letter and location cues were randomly mixed, and separate pure blocks for letter and location tests included only one type of test or the other. These pure blocks should replicate the conditions of Elsley and Parmentier’s procedure almost exactly. If both their findings and ours replicate, then we should find a strong asymmetry when cue type is blocked (similar to the original experiment of Elsely and Parmentier), but not when it is mixed (similar to our Experiment 1). In that case, we would know that this automatic encoding of location with letter information only occurs when participants maintain a consistent attentional set to encode letters.

#### 3.1.1. Participants

We collected these data in 2020–21 online. Participants (initial *N* = 79) were Cardiff University students completing the study for course credits. We did not collect any demographic information. Nine participants were excluded because they acknowledged that they did not do the study in a quiet place without distractions. Participants did not need to meet a standard of response quality to receive credit for taking part, in accordance with the rules governing this sample from the local ethics committee, so we screened data after participation for eligibility. For the accuracy analyses, we set a threshold of minimally 60% correct responses for inclusion in the analyzed data set. This filtering left *N* = 63.

#### 3.1.2. Materials

The letter-in-location materials were presented via a custom script written with *labJS* [27], and deployed via Pavlovia (pavlovia.org). Compared with Experiment 1, we modified our stimuli to better match Elsley and Parmentier’s (2015). We used the same restricted set of letters that they used (D, F, H, J, N, Q, R, T), and displays always included four circled letters.

#### 3.1.3. Design

Our design differed from Elsley and Parmentier’s only in the following ways: (1) we included equal proportions of positive and negative probe trials, with no trials in which both the letter and location at test were new (given that these trials cannot be used to detect binding, they were removed to shorten the experiment); (2) we included two durations, aiming to confirm their finding that differences in delays of 1000 ms and 5000 ms did not change patterns in the data; (3) we introduced one new variable, whether letter and location were mixed within a block and pre-cued, or whether they were separately blocked (and likewise pre-cued). Blocks (mixed, letter tests, locations tests) were randomly ordered for each participant. Each participant completed eight blocks (four mixed blocks, two blocks with letter tests, two blocks with location tests) of 48 trials each (equally split between 1000 and 5000 ms retention, negative and positive probes, with positive probes evenly split between intact, recombined, and new irrelevant feature probes). In mixed blocks, letter and location probes were equally likely. All trial types were randomly ordered within blocks at run time.

#### 3.1.4. Procedure

Working online, participants needed to understand the task independently, without an experimenter present to guide them. We broke instructions into several screens and set minimum presentation times so that participants could not quickly scroll past them. We showed an image of the set of locations where letters might appear so that participants were aware that very small changes in location were not possible, and provided examples of positive and negative probes along with the responses they should elicit. Judging from our initial check on accuracy, these instructions were sufficient to ensure that most of the participants were able to understand the instructions independently.

The trial procedure closely matched that of [2], except for the addition of a pre-cue. Otherwise, the trial events were identical to Elsley and Parmentier’s: a fixation “+” appeared after the cue; then, 500 ms later, four uppercase letter stimuli appeared for 2000 ms. We chose retention intervals of 1000 and 5000 ms, each beginning with a 50 ms noise mask. The probe letter appeared after the retention interval (with no reminder of what content was tested, to increase consistency with Elsley and Parmentier’s design), and remained on-screen until the participant responded. Within a block, the next trial began 1000 ms after the participant’s response. Participants were periodically offered breaks, which could last as long as the participant chose. After all the trials, participants were debriefed and given the chance to withdraw their consent and acknowledge whether they followed instructions about completing the study in an interrupted session.

### 3.2. Results

#### 3.2.1. Accuracy

**Figure 4:**
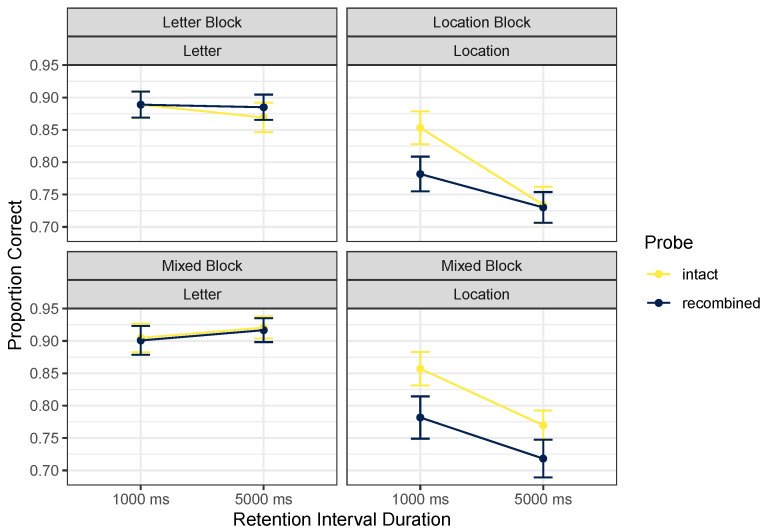
Mean proportions correct, Experiment 2. Error bars mark within-participant standard errors around the mean calculated with the Cousineau–Morey method [25].

As in Experiment 1, we focused on analysis of the positive probes, but descriptive statistics for the full design are given in Table 3. Figure 4 focuses on positive probes. We ran a Cue Content (letters vs. locations) by Probe Type (intact vs. recombined probes) by Retention Interval (1000 vs. 5000 ms) by Block Type (pure vs. mixed) repeated-measures ANOVA on proportions correct (with an arcsine square-root transformation). The best model included main effects of cue content and retention interval, along with an interaction between cue content and retention interval, *BF* = $8.5\times 10^{22}$. Excluding the effect of probe type, which would indicate that incidental binding took place, was only marginally favored, *BF* = 1.57. Similarly, excluding the crucial interaction between cue content and probe type was only narrowly favored, *BF* = 2.52. However, judging from Figure 4, any interaction observed here would support the opposite asymmetry to that reported by [2], namely that binding may have been more likely to be remembered during location tests than during letter tests. Excluding the effect of pure vs. mixed blocks of letter and location tests was favored by a factor of 7.15; excluding any interactions with the block factor was even more clearly favored. This suggests that whatever binding between letter and location may be happening does not require instantiating an attentional set for a long period: it is apparently as likely to happen when switching between letter and location tests as when focusing for a block of trials on only one kind of test.

The pattern of findings here differs substantially from that of [2]. If we consider inclusion of the marginal interaction between probe type and cue content, the interpretation has to be that there is a binding effect in the location trials, where intact > recombined probes (indicating that irrelevant letter information was maintained when only location information was relevant), with no such pattern in the letter trials (indicating that irrelevant location information was *not* maintained when only letter information was relevant). The interaction between cue content and retention interval (which was favored; *BF* = 57.13) appears from Figure 4 to reflect an effect of retention interval on location trials only, with accuracy falling at the longer retention interval. This effect of retention interval in these ranges is also inconsistent with Elsley and Parmentier’s findings.

#### 3.2.2. Response Times

**Figure 5:**
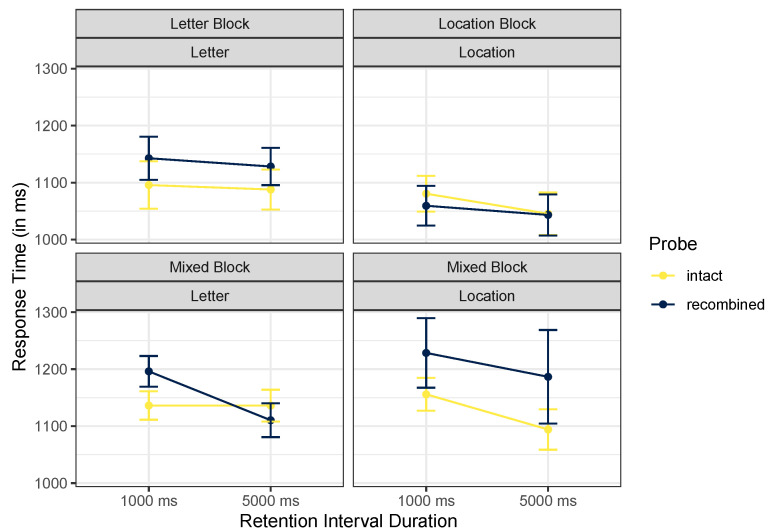
Mean trimmed reaction times, Experiment 2. Error bars mark within-participant standard errors around the mean calculated with the Cousineau–Morey method [25].

We ran corresponding analyses on log-transformed response times, excluding incorrect responses. Table 4 shows descriptive values for all conditions, and Figure 5 focuses on the positive probe conditions meant to identify incidental binding.

The best model included a main effect of block only, *BF* = 13.44. Excluding probe type was favored only by a factor of 1.57. The ANOVA did not support any interactions in these data, including the crucial cue content by probe type interaction. Exclusion of this interaction was strongly preferred, *BF* = 200.29.

### 3.3. Discussion

With Experiment 2, we attempted to replicate the key supporting finding for strong binding asymmetry, namely that locations appear to be encoded and maintained in a recognition task where only letters are relevant at test, but letters are not maintained when only locations are relevant at test. We did not observe any evidence for this pattern in accuracies or response times, regardless of whether letter and location tests were blocked or mixed and pre-cued. If anything, there was more support for obligatory letter memory during location tests. This reverse asymmetry has been demonstrated previously, but with spatially distributed aural stimuli [28]. We consider the implications of our findings further in the General Discussion.

## 4. General Discussion

We detailed two studies, aimed at examining the strong asymmetry between verbal and spatial information in the maintenance of letter–location combinations, as previously demonstrated by [2], showing locations to be bound automatically to letters but not the other way round. In Experiment 1, examining the effect of pre-cues and retro-cues on accuracy and speed of response, we found no evidence for the strong asymmetry hypothesis. Intact probes were recognized somewhat better than recombined probes, but this did not vary between letter- and location-focused trials. This also did not vary as a function of whether the information was cued before or after encoding. Regarding reaction times, there was no evidence to indicate the occurrence of incidental binding in either the letter or location task, and strong evidence against the asymmetry hypothesis. Experiment 2 aimed to replicate the work of [2] more closely. The retro-cues were dropped and half of the trials were presented in single-focus (letter or location only) and thus pure blocks, as was the case in the study by [2], and the other half of the trials in mixed blocks as in Experiment 1. Nevertheless, this closer replication also failed to demonstrate the predicted pattern. The results of this second study also indicate that there was no effect of mixing the trial types within a block. Contrary to the expected direction of binding asymmetry, analyses of accuracy uncovered no evidence of location-to-letter binding; if anything, binding of letter information to locations was more plausible (i.e., an asymmetrical pattern in the opposite direction). Regarding response times, again, the important interaction of cue content by probe type was not supported by the data, indicating no occurrence of differential binding. To summarize, none of the data collected in the current studies support a strong binding asymmetry in the direction that [2] and others (e.g., [29]) have documented, whereby locations bind to letters when letters are the focus of the task, without the opposite being true.

Where does this leave us in understanding whether encoding location may be obligatory when encoding identity, and the potential important asymmetry in terms of working memory resources involved in letter–location combinations? Though this is most readily interpreted as a straightforward non-replication, we think there have been enough published examples [2,15,29] of differences in feature binding based on whether location or identity is the focus of the task that our findings should not be taken as a simple repudiation of prior research. However, it should also be noted that others have also found that locations are not necessarily bound with identity [30,31], which suggests that, more broadly, the encoding and maintenance of features and their relation to location is likely to be quite flexible. Although we attempted to keep our procedure in Experiment 2 very close to Elsley and Parmentier’s, there must be moderating factors that, if we were fully aware of them, could predict when features are encoded together. Alternatively, there could be individual differences in participants’ approach to these tasks that affect some samples quite strongly; given our results, we think that incidental location-to-letter binding is unlikely to be a phenomenon that all participants experience to some degree [32]. With the two studies presented here, we rule out some potential moderating factors. First, we did not observe systematic differences in incidental binding when the target feature was known during encoding or only afterward, much as [28] found with spatially distributed aural materials. This means that simply knowing which feature will be tested during encoding is not sufficient to induce incidental binding. Furthermore, blocking pre-cues so that participants can establish a longer-term attentional set for encoding one feature and perhaps inhibiting the other is also not sufficient for inducing incidental binding of location to letters. It remains possible that prior knowledge of the test feature and establishing an attentional set for encoding this feature is necessary to observe the location-to-letter binding asymmetry, but these considerations alone do not guarantee that it will occur.

Some readers may attribute our lack of a strong asymmetrical pattern to our choice to conduct Experiment 2 online. We think that this is mistaken for a few reasons. First, we did begin to conduct Experiment 2 in person prior to the onset of pandemic restrictions in early 2020, but the small sample (*N* = 12) that we acquired also showed no evidence of the asymmetric pattern reported by [2]. Moreover, informal comparison of the pre-cue results of our Experiment 1 (which was the condition most comparable to Elsley and Parmentier’s design and was conducted in the laboratory) and Experiment 2 gives no reason to presume that the participants in our online sample were inattentive or did not know what to do: accuracies were quite comparable. The accuracy rates observed in Experiment 2 are also similar to those that [2] reported. Altogether, there is no reason to think that the online sample was inadequate for the purpose of replicating Elsley and Parmentier’s findings.

Given that our experiments did not reveal the same patterns as previously observed, how should researchers proceed to discover and solidify assumptions about the incidental binding of letter identity to location? We suspect that implicit recognition paradigms such as these present too much potential for uncontrolled and unaccountable variability, both across participants and across administrations. Because the measure is implicit, it is difficult to feel confident about whether participants understood how to judge the probes. How often might they simply forget which task they are doing, or become unsure about whether to take the irrelevant feature into account with their response? These problems pose particular difficulties for interpreting accuracy, because interpretation depends on why participants make errors on recombination trials. These errors are only interesting in as much as we can be confident that participants always understood what they were meant to be judging. We think that it would be most beneficial to assess incidental binding using a variety of methods for convergence and focusing either on responses times in samples where accuracy is nearly perfect or making use of rare explicit tests such as those used to illustrate attribute amnesia [33] to increase confidence about what exactly accuracy reveals.

Even outside the realm of binding, location appears to be special for working memory. Ref. [34] used a parity judgment task to investigate the well-documented Spatial Numerical Association of Response Codes (SNARC; Dehaene, Bossini, & Giraux, 1993, as cited in [34]) effect. This effect entails, on a basic level, an association of smaller numbers with the left side and larger numbers with the right side, seemingly reflecting a number line. This direction of association is reversed in those whose language reads right to left, though, interestingly, in bilingual speakers of one of each of these language types, it has been demonstrated that priming with one type of language can incite a change in the direction of the effect to match the direction of reading (Shaki & Fischer, 2008, as cited in [34]). In response to this strange flexibility, van Dijck and Fias formulated and tested the notion that it is changeable ordinal information from the sequence in which the items are presented, as maintained in working memory, rather than unchanging long-term memory that underlies the SNARC effect. Indeed, they found evidence to support this in memory for not just numbers, but verbal information too (fruit and vegetable names). This finding is intriguing when paired with the findings of a study conducted by [35] on location-independent feature binding. In this study, participants performed better in visual working memory tasks when the sequentially presented items were presented in different locations than when they were presented all in the same location, an effect that the authors attributed to encoding interference. These studies together could be taken to indicate that in the absence of spatial differences, working memory will attempt to assign spatial information to the unoriented information in a sequence to aid remembering. However, the cognitive system’s efforts to impose spatial information on unoriented items may not be as effective as when items already possess spatial associations in the real world, hence the finding that memory for items presented all in one location is not as good. Perhaps the reason that this assignment of spatial information benefits the working memory system is that it increases the distinctness of the items on a spatial spectrum, which may reduce encoding interference, aligning with Schneegans et al.’s conclusions. When verbal information already has associated spatial information in the world, as is the case in verbal–spatial binding tasks, the working memory system need not assign spatial information to the items. This may contribute to why locations sometimes get their free ride, and could be something to consider going forward, even in light of the current study’s failure to replicate the expected pattern of binding.

### Conclusions

In two experiments that conceptually or nearly replicated Elsley and Parmentier’s (2015) letter–location recognition procedure, we did not observe the asymmetric pattern of binding that they reported. We conclude that this pattern is not likely to be robust across participants and may be dependent on circumstantial factors. We maintain that, on balance, other findings illustrating interplay between letter and location information still strongly suggest that features of various types can and often are encoded in an integrated manner. However, our findings caution against presuming that there are immutable regularities in how identity features and their locations are encoded and maintained. Our results suggest that verbal–spatial binding asymmetry should not yet be allowed to influence current working memory models, as it is clear that we cannot yet extract general principles about asymmetric binding that might be used to restrict working memory theories.

## Figures and Tables

**Table 1:** Mean proportion correct (with standard deviations) per cue timing, cue content, probe type, and set size, Experiment 1.

Cue Timing	Cue Content	Probe	Set Size	Mean	SD
Pre-Cue	Letters	Intact	3	0.97	0.07
			5	0.95	0.09
		New-Both	3	0.97	0.07
			5	0.96	0.08
		New-Relevant	3	0.95	0.12
			5	0.94	0.11
		Recombined	3	0.97	0.06
			5	0.93	0.12
	Locations	Intact	3	0.80	0.18
			5	0.75	0.22
		New-Both	3	0.86	0.19
			5	0.82	0.19
		New-Relevant	3	0.81	0.18
			5	0.76	0.24
		Recombined	3	0.74	0.25
			5	0.74	0.21
Retro-Cue	Letters	Intact	3	0.96	0.10
			5	0.93	0.10
		New-Both	3	0.93	0.17
			5	0.94	0.14
		New-Relevant	3	0.96	0.10
			5	0.94	0.10
		Recombined	3	0.93	0.12
			5	0.91	0.16
	Locations	Intact	3	0.78	0.18
			5	0.75	0.18
		New-Both	3	0.81	0.20
			5	0.82	0.18
		New-Relevant	3	0.80	0.16
			5	0.72	0.21
		Recombined	3	0.67	0.25
			5	0.74	0.22

*Note.* N = 41.

**Table 2:** Mean trimmed response times (with standard deviations) per cue timing, cue content, probe type and set size, Experiment 1.

Cue Timing	Cue Content	Probe	Set Size	Mean	SD
Pre-Cue	Letters	Intact	3	1298	332
			5	1500	497
		New-Both	3	1401	478
			5	1623	867
		New-Relevant	3	1417	519
			5	1576	548
		Recombined	3	1339	413
			5	1480	382
	Locations	Intact	3	1503	646
			5	1656	771
		New-Both	3	1258	332
			5	1384	486
		New-Relevant	3	1362	433
			5	1429	525
		Recombined	3	1585	627
			5	1613	681
Retro-Cue	Letters	Intact	3	1368	344
			5	1473	445
		New-Both	3	1528	537
			5	1665	695
		New-Relevant	3	1484	696
			5	1679	647
		Recombined	3	1379	389
			5	1526	336
	Locations	Intact	3	1572	526
			5	1703	659
		New-Both	3	1384	428
			5	1554	505
		New-Relevant	3	1405	381
			5	1553	697
		Recombined	3	1592	639
			5	1720	688

*Note.* N = 41.

**Table 3:** Mean proportion correct (with standard deviations) per cue content, probe type, and retention interval, Experiment 2.

Block	Cue Content	Probe	Retention Interval	Mean	SD
Letter	Letter	intact	1000 ms	0.89	0.17
			5000 ms	0.87	0.20
		newLetter	1000 ms	0.95	0.10
			5000 ms	0.92	0.14
		newLocation	1000 ms	0.88	0.16
			5000 ms	0.91	0.15
		recombined	1000 ms	0.89	0.18
			5000 ms	0.88	0.17
Location	Location	intact	1000 ms	0.85	0.22
			5000 ms	0.73	0.25
		newLetter	1000 ms	0.79	0.24
			5000 ms	0.70	0.28
		newLocation	1000 ms	0.85	0.16
			5000 ms	0.81	0.13
		recombined	1000 ms	0.78	0.25
			5000 ms	0.73	0.20
Mixed	Letter	intact	1000 ms	0.90	0.19
			5000 ms	0.92	0.15
		newLetter	1000 ms	0.91	0.10
			5000 ms	0.90	0.13
		newLocation	1000 ms	0.89	0.19
			5000 ms	0.85	0.19
		recombined	1000 ms	0.90	0.18
			5000 ms	0.92	0.15
	Location	intact	1000 ms	0.86	0.21
			5000 ms	0.77	0.21
		newLetter	1000 ms	0.76	0.23
			5000 ms	0.69	0.30
		newLocation	1000 ms	0.82	0.13
			5000 ms	0.80	0.18
		recombined	1000 ms	0.78	0.30
			5000 ms	0.72	0.27

*Note.* N = 63.

**Table 4:** Mean trimmed response times (with standard deviations) per cue timing, cue content, probe type and set size, Experiment 2.

Block	Cue Content	Probe	Retention Interval	Mean	SD
Letter Block	Letter	intact	1000 ms	1096	421
			5000 ms	1088	361
		newLetter	1000 ms	1199	370
			5000 ms	1136	359
		newLocation	1000 ms	1071	279
			5000 ms	1042	287
		recombined	1000 ms	1143	462
			5000 ms	1129	359
Location Block	Location	intact	1000 ms	1079	316
			5000 ms	1042	308
		newLetter	1000 ms	1062	316
			5000 ms	1080	397
		newLocation	1000 ms	1061	287
			5000 ms	985	263
		recombined	1000 ms	1061	320
			5000 ms	1044	290
Mixed Block	Letter	intact	1000 ms	1137	282
			5000 ms	1137	318
		newLetter	1000 ms	1186	282
			5000 ms	1182	346
		newLocation	1000 ms	1219	342
			5000 ms	1155	407
		recombined	1000 ms	1197	379
			5000 ms	1109	337
	Location	intact	1000 ms	1156	385
			5000 ms	1095	315
		newLetter	1000 ms	1232	568
			5000 ms	1082	344
		newLocation	1000 ms	1114	342
			5000 ms	1067	310
		recombined	1000 ms	1224	645
			5000 ms	1190	825

*Note.* N = 63.

## Data Availability

Data and materials are available at https://osf.io/c9xzh/.
